# Regulation of myogenesis and adipogenesis by the electromagnetic perceptive gene

**DOI:** 10.1038/s41598-023-48360-6

**Published:** 2023-12-01

**Authors:** Jangsun Hwang, Hae Woon Jung, Kyung Min Kim, Daun Jeong, Jin Hyuck Lee, Jeong-Ho Hong, Woo Young Jang

**Affiliations:** 1 Department of Orthopedic Surgery, College of Medicine, Korea University, 73 Korea-ro, Seongbuk-gu, Seoul, 02841 Republic of Korea; 2 Department of Pediatrics, Kyung Hee University Medical Center, Seoul, Republic of Korea; 3 Department of Life Sciences, School of Life Sciences and Biotechnology, Korea University, Seoul, 02841 Republic of Korea; 4 Institute of Nano, Regeneration, and Reconstruction, College of Medicine, Korea University, 73 Korea-ro, Seongbuk-gu, Seoul, 02841 Republic of Korea

**Keywords:** Biotechnology, Cell biology, Diseases, Health care

## Abstract

Obesity has been increasing in many regions of the world, including Europe, USA, and Korea. To manage obesity, we should consider it as a disease and apply therapeutic methods for its treatment. Molecular and therapeutic approaches for obesity management involve regulating biomolecules such as DNA, RNA, and protein in adipose-derived stem cells to prevent to be fat cells. Multiple factors are believed to play a role in fat differentiation, with one of the most effective factor is Ca^2+^. We recently reported that the electromagnetic perceptive gene (EPG) regulated intracellular Ca^2+^ levels under various electromagnetic fields. This study aimed to investigate whether EPG could serve as a therapeutic method against obesity. We confirmed that EPG serves as a modulator of Ca^2+^ levels in primary adipose cells, thereby regulating several genes such as CasR, PPARγ, GLU4, GAPDH during the adipogenesis. In addition, this study also identified EPG-mediated regulation of myogenesis that myocyte transcription factors (CasR, MyoG, MyoD, Myomaker) were changed in C2C12 cells and satellite cells. In vivo experiments carried out in this study confirmed that total weight/ fat/fat accumulation were decreased and lean mass was increased by EPG with magnetic field depending on age of mice. The EPG could serve as a potent therapeutic agent against obesity.

## Introduction

Obesity is a condition of global concern that also leads to various other problems such as cardiovascular problems, diabetes mellitus, hypertension, and stroke^1^. In particular, there has been a steady increase in childhood obesity in the Western and developing countries over the last two decades. It has become a global public health concern and is associated with a range of short- and long-term health complications^2^. To prevent childhood obesity, there is a need to focus on a combination of diet, exercise, and/or behavioral modifications. Muscle gain and fat loss should serve as the top priority in efforts to treat obesity; however, since the patient population in case of childhood obesity includes children, these treatments have to be restricted. Thus, currently, there is no effective treatment for childhood obesity. Various factors such as age, gender, and food consumption habits serve as the main causative factors of obesity, which is characterized by an increase in adipose mass, hypertrophy, hyperplasia of fat cells, and differentiation of multipotent mesenchymal stem cells^3^. Multiple factors are involved in fat differentiation from mesenchymal stem cells, including peroxisome proliferator-activated receptor gamma (PPARγ), glucose transporter-4 (GLU4), and calcium (Ca^2+^).

In a previous study, we confirmed that synthetic biological devices serve as an “artificial biological switch” to allow non-invasive control of cell fate and function^4^. Activation of EPG by electromagnetic field has been successfully reported in in vivo and in vitro models, by means of manipulation of [Ca^2+^]i^4, 5^. Normal cells require high external Ca^2+^ concentrations to trigger cell proliferation^6, 7^. The mechanism for Ca^2+^ entry is store-operated calcium entry (SOCE), which involves voltage-dependent Ca^2+^ channels (expressed in mesenchymal cells, cancer stem cells, lymphocytes, and breast cancer cells) that are essential for gene regulation and cell proliferation. According to El Boustany et al.^8^ SOCE is a more important factor for cell proliferation than the functions of external Ca^2+^ and channels. Additionally, cell proliferation rates depended on the relative balance between Ca^2+^ influx and the CaSR. Therefore, regulation of [Ca^2+^]i by means of EPG activation enables the regulation of cell proliferation and fate, especially in ADSCs and myoblasts.

Among the various factors involved in transcription, Ca^2+^ plays important roles in different stages of cell development and fate. However, owing to its complicated roles in cells, there is little research on therapeutic treatments involving Ca^2+^.

Calcium ions are involved in several cellular functions, including differentiation, proliferation, apoptosis, and cell death^9–12^. It has a dual effect on adipogenesis: an inhibitory effect in the early stages but a stimulating effect in the later stages^4–6^. Ca^2+^ stimulates triglyceride (TG) metabolism by controlling lipolysis and lipogenesis^13^. In addition, calreticulin and calcium-sensing receptor (CaSR) have been reported to regulate adipogenesis in opposite ways, through regulation of PPARγ2. Intracellular calcium is fundamental to the early and late stages of myoblast differentiation. The muscle-specific transcription factors myogenin and myocyte enhancer factor 2 depend on the generation of Ca^2+^ signals^14^. In myoblasts, elevations in [Ca^2+^]i levels are a result of Ca^2+^ release from the endoplasmic reticulum and/or Ca^2+^ entry through the plasma membrane^15^.

A study performed calcium imaging in electromagnetic perceptive gene (EPG)-expressing mammalian cells and cultured neurons isolated from *Kryptopterus bicirrhis* (glass catfish) and demonstrated that remote activation by means of an electromagnetic field significantly increased the [Ca^2+^]i concentrations^5^. In this study, we investigated the functional mechanism of EPG under magnetic field (MF), possibly by enhancing the influx of calcium into cells, thereby increasing [Ca^2+^]i levels, which in turn regulated genes that affect the differentiation of mesenchymal stem cells to adipocytes and myoblasts to fat and muscles in vitro and in vivo. In addition, we investigated how adipogenesis and myogenesis are regulated by Ca^2+^ in response to EPG with a MF.

## Results

### Regulation of target protein expression by EPG under the control of a calcium ion-sensitive promoter

**Figure 1:**
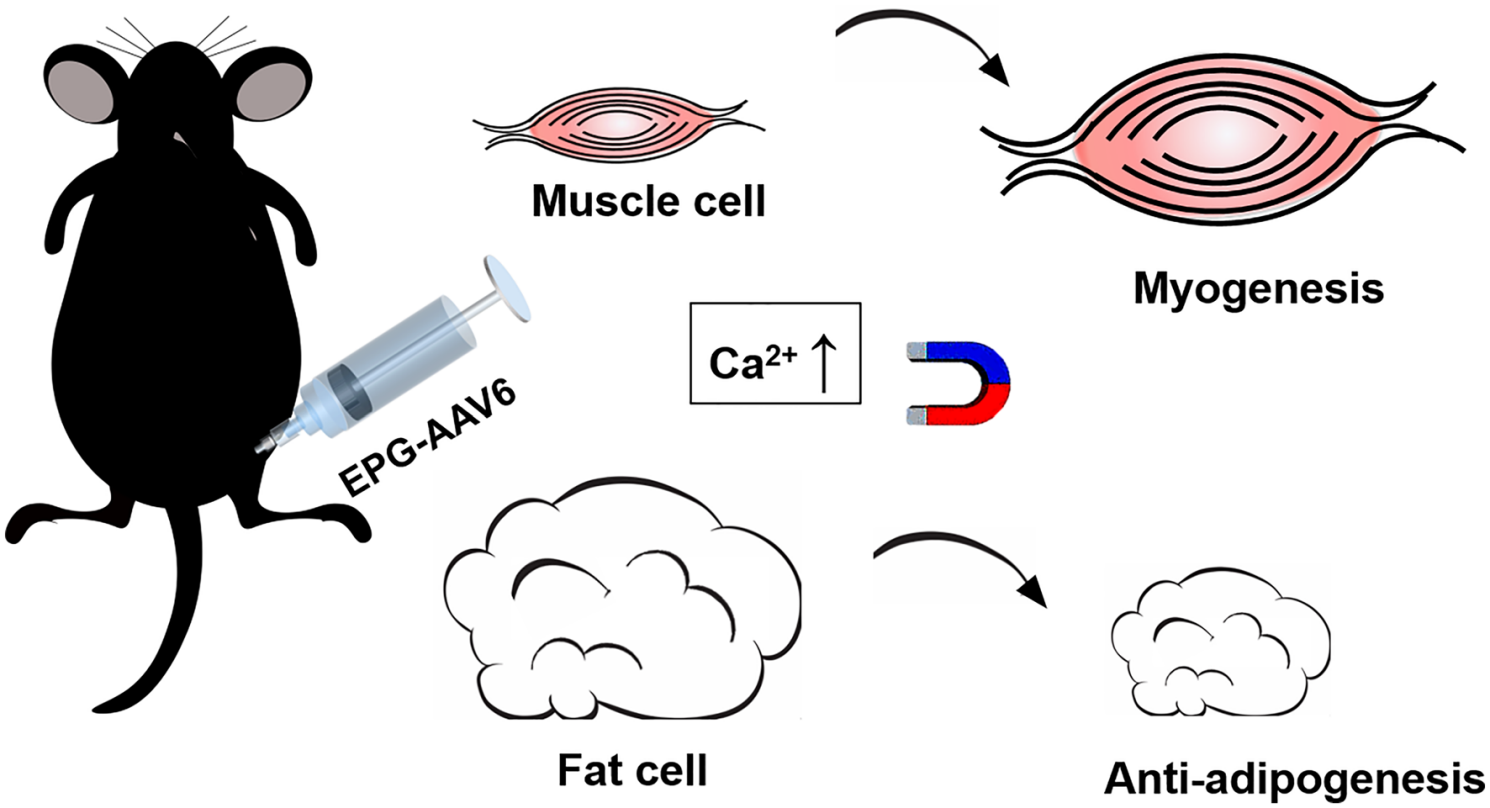
Schematic representing the mechanisms by which EPG activated by a magnet field regulates myogenesis and adipogenesis.

Controll of [Ca^2+^]i by means of EPG activation with magnetic field enables the regulation of cell proliferation and fate, especially in ADSCs and myoblasts (Fig. 1). First, we assayed the expression of EPG in HEK-293T cells by tagging it with GFP (Supporting Fig. 1) and found it to be approximately 40%^4^ and localized in the cellular membrane. [Ca^2+^]i has been shown to be regulated when EPG is activated by a MF or electromagnetic field in various cells, such as kidney and nerve cells^5^. Therefore, we also confirmed that [Ca^2+^]i is managed by EPG + MF in HEK-293T cells (Supporting Fig. 2A,B).

Upon Fura-2AM staining, the fluorescence signal intensity increased by 20 ± 5% in HEK293T^EPG^ with a MF, as compared to that with no magnetic field (Supporting Fig. 2A,B). [Ca^2+^]i changes were observed within 300 s, depending on the cell-type^4, 5^. Based on these results, we deduced that [Ca^2+^]i induces cell development by regulating target gene expression and protein synthesis. To confirm this, we adopted a calcium-sensitive promoter that enabled control of the target protein (RFP) to reveal the functionality of EPG. c-Fos is a proto-oncogene that is rapidly induced by a wide variety of stimuli, including polypeptide growth factors, phorbol esters, and calcium ion fluxes^16^ (Supporting Fig. 2C, left). Higher levels of [Ca^2+^]i may stimulate the c-Fos promoter to enhance RFP expression (Supporting Fig. 2C, right).

As shown in Supporting Fig. 2D–F, the EPG with MF group showed higher RFP expression (25 ± 5%) after 4 h of incubation, while the EPG without MF and RFP with MF groups showed 9 ± 1% and 10 ± 1% RFP expression, respectively. Thus, EPG activation using a MF enhanced the expression of the target protein (RFP), which is regulated by calcium influx (Supporting Fig. 2D–H).

### EPG expression in C2C12 cells, hADSCs, and satellite cells

Upon transfection of mouse myoblast cells (C2C12 cells), hADSCs, and satellite cells with EPG-RFP (Supporting Fig. 3A,B), it was found that the percentage of EPG-expressing cells in these three cells lines was lower than that in HEK-293T cells^4^.

It is known that uncontrolled Ca^2+^ influenced cell proliferation and apoptosis^17^; Choi et al*.* suggested that very high [Ca^2+^]i levels promoted cell death through necrosis, while lower [Ca^2+^]i increased cell death through apoptosis^18, 19^. C2C12 cells and hADSCs were observed after transfection with EPG-RFP. hADSC^EPG^ showed no sign of cytotoxicity after a short period of magnetic field; however, long-term magnetic field (24 h) led to a cell death of 20 ± 2% (Supporting Fig. 4A). On the other hand, C2C12^EPG^ showed a decrease in cell proliferation of 20 ± 2% on treatment with EPG only, 23 ± 2% after 10 min of MF, and 25 ± 3% after 24 h of MF (Supporting Fig. 4B). Additionally, after 3 days of transfection, ADSC^EPG^ remained 40% (Supporting Fig. 4C). Thus influencing the cell fate in both cells, which is regulated by [Ca^2+^]i.

### Calcium ion mapping

Excitable cells such as myoblasts and ADSCs are known to be sensitive to Ca^2+^. Many studies have shown that myogenesis and adipogenesis were related to high [Ca^2+^]i levels^20–23^. In the next step of the study, we checked for calcium influx in myoblasts and hADSCs. Both cell lines were transfected with EPG and then treated with growth medium for 2 days. Next, EPG^+^ cells were stained with Fura-4AM, following which 10 µL of magnetic beads (~ 50 mT) were directly applied to the cells, and the cells were mapped (Supporting Fig. 5). Non-transfected cells showed no change in fluorescence upon application of the magnetic beads (Supporting Fig. 5A–C). On the other hand, hADSC^EPG^ and C2C12^EPG^ cells showed increases of 51 ± 5% and 30 ± 3% in the fluorescence signal, respectively, upon application of magnetic beads (Supporting Fig. 5B–D).

### Evaluation of gene expression

[Ca^2+^]i regulates cell differentiation, especially that of primary cells. For example, higher [Ca^2+^]i levels act as a transcriptional factor that induces myoblasts to differentiate into muscle cells^12^. To verify this, we determined the mRNA expression in C2C12 cells during the differentiation stages.

**Figure 2:**
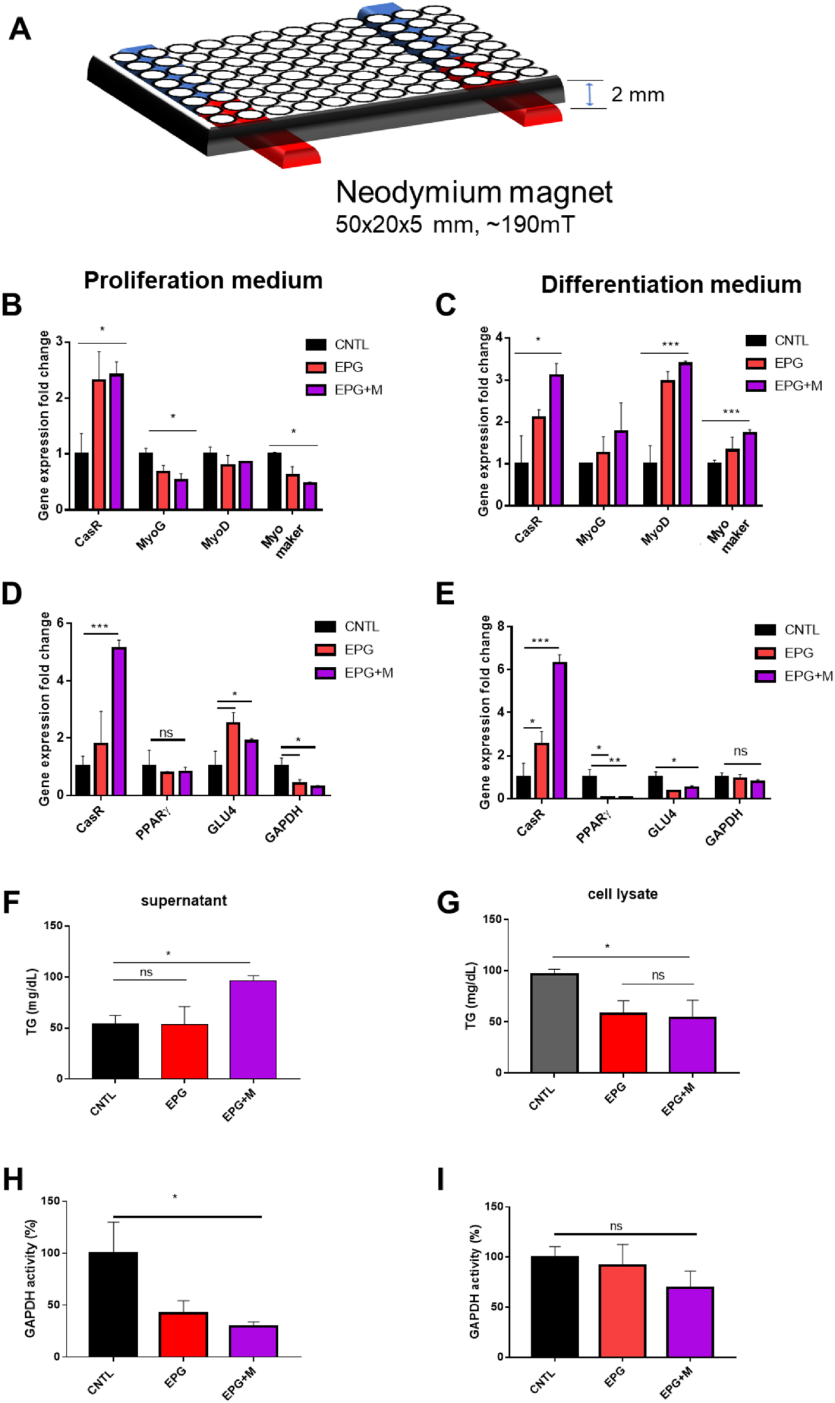
Evaluation of gene expression. (**A**) Illustration of the experimental setup (Neodymium magnet were placed under 96 well, 50 × 20 × 5 mm, ~ 196 mT). (**B**) Gene expression levels of MyoG, MyoD, and Myomaker in EPG-transfected C2C12 cells cultured in growth medium and (**C**) differentiation medium. (**D**) Gene expression levels of CaSR, PPARγ2, GLU4, and GAPDH in EPG-transfected hADSCs cultured in growth medium and (**E**) differentiation medium. (**F**) Evaluation of TG in supernatant and (**G**) cell lysate in ADSCs^EPG^. (**H**) GAPDH activity in ADSCs^EPG^ cultured in growth medium and (**I**) differentiation medium. The qPCR data was normalized to the levels of α-actin; CNTL, no treatment group; magnet = ~ 196 mT; magnetic field was carried out for 1 day; n = 3).

All experiments were performed a customized magnetic environmental set up (Fig. 2A, Table 1). There was a 2.5 ~ threefold increase in the mRNA expression of CaSR (which regulates the proliferation and apoptosis of human vascular smooth muscle cells^24^) in C2C12^EPG^ cells, after magnetic field during both medium. In proliferation medium, the mRNA levels of MyoG, MyoD, and Myomaker did not increase (Fig. 2B) (*P* < 0.05). In differentiation medium, there was 1.1-fold, 3.3-fold, and 1.7-fold increase in the mRNA expression of MyoG, MyoD, and Myomaker with EPG + FM respectively, which play major roles in regulating muscle differentiation^25, 26^ (Fig. 2C). This suggests that muscle differentiation is accelerated by EPG with a MF in myoblasts. Based on these results, we concluded that EPG + MF treatment enabled manipulation of calcium influx, which regulates mRNA expression, resulting in enhanced myogenesis.

**Table 1:** Primer pairs.

Protein	Gene	Forward	Reverse
Murine MyoD	MyoD	5’ GCCGGTGTGCATTCCAA 3’	5’ CACTCCGGAACCCCAACAG 3’
Murine MyoG	MyoG	GACCTGATGGAGCTGTATGAG	CTGAAGGTGGACAGGAAGG
Murine Myomaker	MYMK	CTGAGCTCCCAAGACATGAG	CCAATCTCTCCTTCCTCTGG
Murine MCK	MCK	CACCTCCACAGCACAGACAG	ACCTTGGCCATGTGATTGTT
Murine MHC	MHC1	ACAAGCTGCGGGTGAAGAG	CAGGACAGTGACAAAGAACG
Murine GAPDH	GAPDH	GTTGTCTCCTGCGACTTCA	GGTGGTCCAGGGTTTCTTA
Human PPARγ2	PPARγ2	CTATTGACCCAGAAAGCGAT	CGTAATGTGGAGTAGAAATGC
Human GLUT4	GLUT4	AGGATCGGTTCTTTCATCTTCGC	GTTCCCCATCTTCGGAGCCTA
Human CasR	CasR	TCTCAAATCAAGGCCGGAGT	GCTGTTTATCTCCTCTATGGCAA
Human Beta actin	ATP5PB	TCGAGTCGCGTCCACC	GGGAGCATCGTCGCCC

In hADSC^EPG^ cells, the expression of CasR mRNA increased 5 ± 0.3-fold and 6 ± 0.3-fold upon EPG + MF treatment in the proliferation and differentiation medium, respectively. The mRNA expression of GLU4, an insulin-responding glucose transporter^27^, increased (1.7 ± 0.2-fold) in proliferation medium while that of GAPDH (0.2 ± 0.01-fold) decreased (Fig. 2D). In contrast, the mRNA expression levels of PPARγ (glitazone reverse insulin resistance receptor) and GLU4 decreased by 0.03 ± 0.007-fold and 0.5 ± 0.1-fold, respectively, in differentiation medium (Fig. 2E). Additionally, PPARγ and GLU4 mRNA levels were downregulated upon EPG treatment alone. Similar to GAPDH mRNA expression, GAPDH activity reduced upon EPG with a MF treatment in proliferation medium, while there was no change in differentiation medium (Fig. 2D,E). There was an increase in the TG content (which is regulated by PPARγ) in the supernatant but a decrease in that in the cell lysate upon EPG + MF treatment (Fig. 2F,G). In addition, in proliferation medium, MF decreased GAPDH activity (48 ± 6%), as compared to that in the control group (Fig. 2H,I). Based on these results, we deduced that EPG lowered the expression of PPARγ and GLU4, which in turn induced less TG synthesis, resulting in less fat accumulation in adipose cells.

### Regulation of adipogenesis by EPG + MF

**Figure 3:**
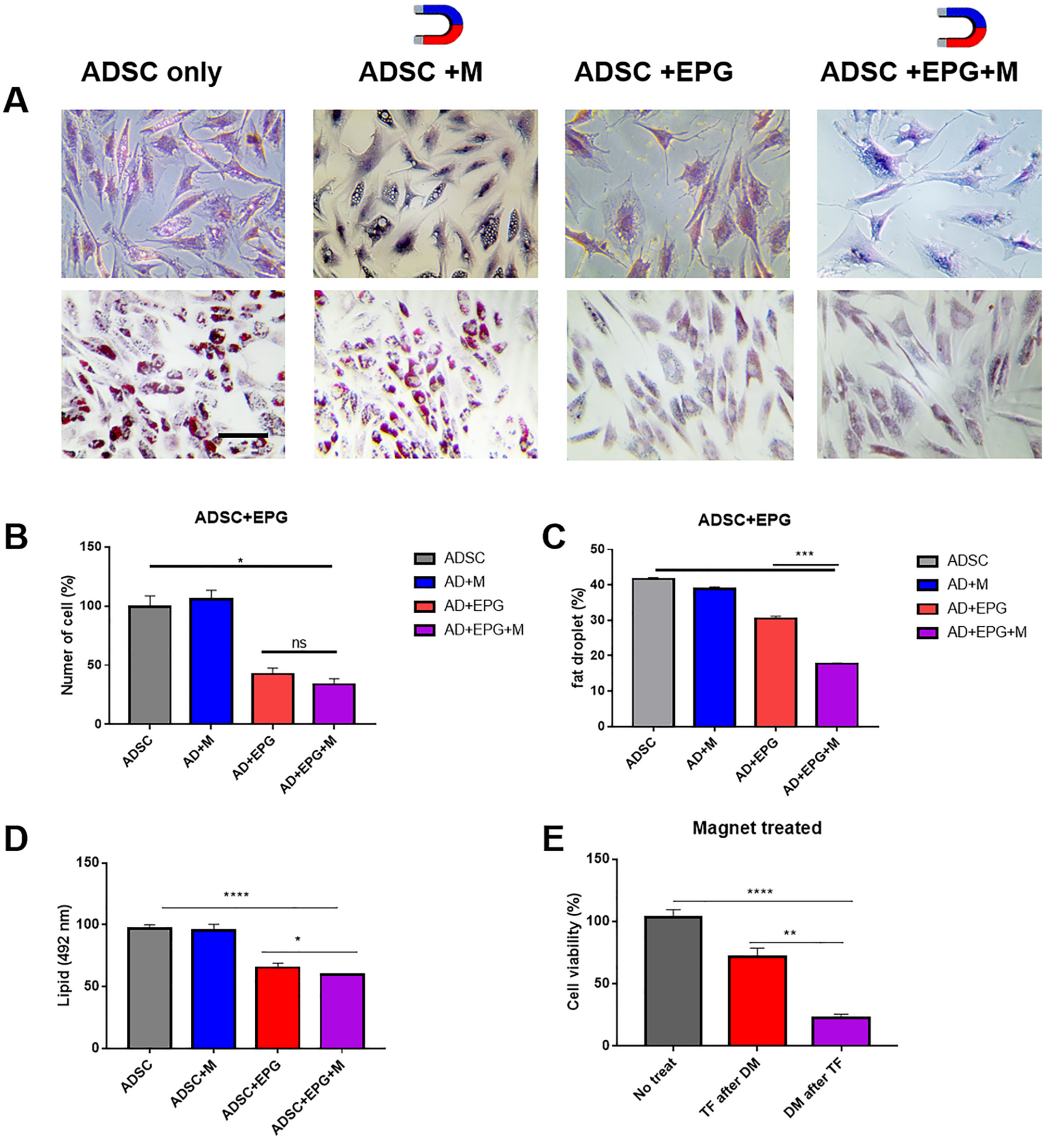
Regulation of adipogenesis by EPG. (**A**) Lipid droplet formation, as observed by means of Oil Red O staining (scale bar = 20 µm, M = magnetic field). (**B**) Total cell count and (**C**) total fat droplet count after EPG with or without magnetic field. (**D**) Quantification of lipid after EPG with or without magnetic field. (**E**) Cell viability before/after cell differentiation (Neodymium magnet was placed under the 96 wells, treatment was carried out with a ~ 196 mT magnet for 2 d, followed by 10 d of culture; DM, differentiation; TF, transfection; n = 3; M, magnetic field).

MF-activated EPG influenced calcium influx in ADSCs, leading to reduced lipid synthesis, by regulating several genes such as CaSR, GLU4, PPARγ, and GAPDH. To confirm this, we monitored fat droplet formation after adipocyte differentiation, using Oil Red O staining (Fig. 3A). After 10 d of incubation, the EPG + MF and EPG only groups showed decreased cell numbers by 48 ± 3% and 60 ± 5%, respectively (Fig. 3B). There was a significant reduction in the number of lipid droplets as compared to those in the control group: 25 ± 5% for the EPG only group and 55 ± 5% for the EPG + MF group (Fig. 3C). Additionally, the total lipid content decreased by > 35% (Fig. 3D). EPG expression after adipocyte differentiation leads to a great amount of cell death (Fig. 3E), and calcium influx is a critical factor in regulating cell viability and adipogenesis. Therefore, EPG activated by a MF not only regulates lipid formation in each cell but also controls cell fate, which influences body fat formation; this particular effect of EPG can be used to treat obesity.

### Regulation of myogenesis upon treatment with EPG + MF

**Figure 4:**
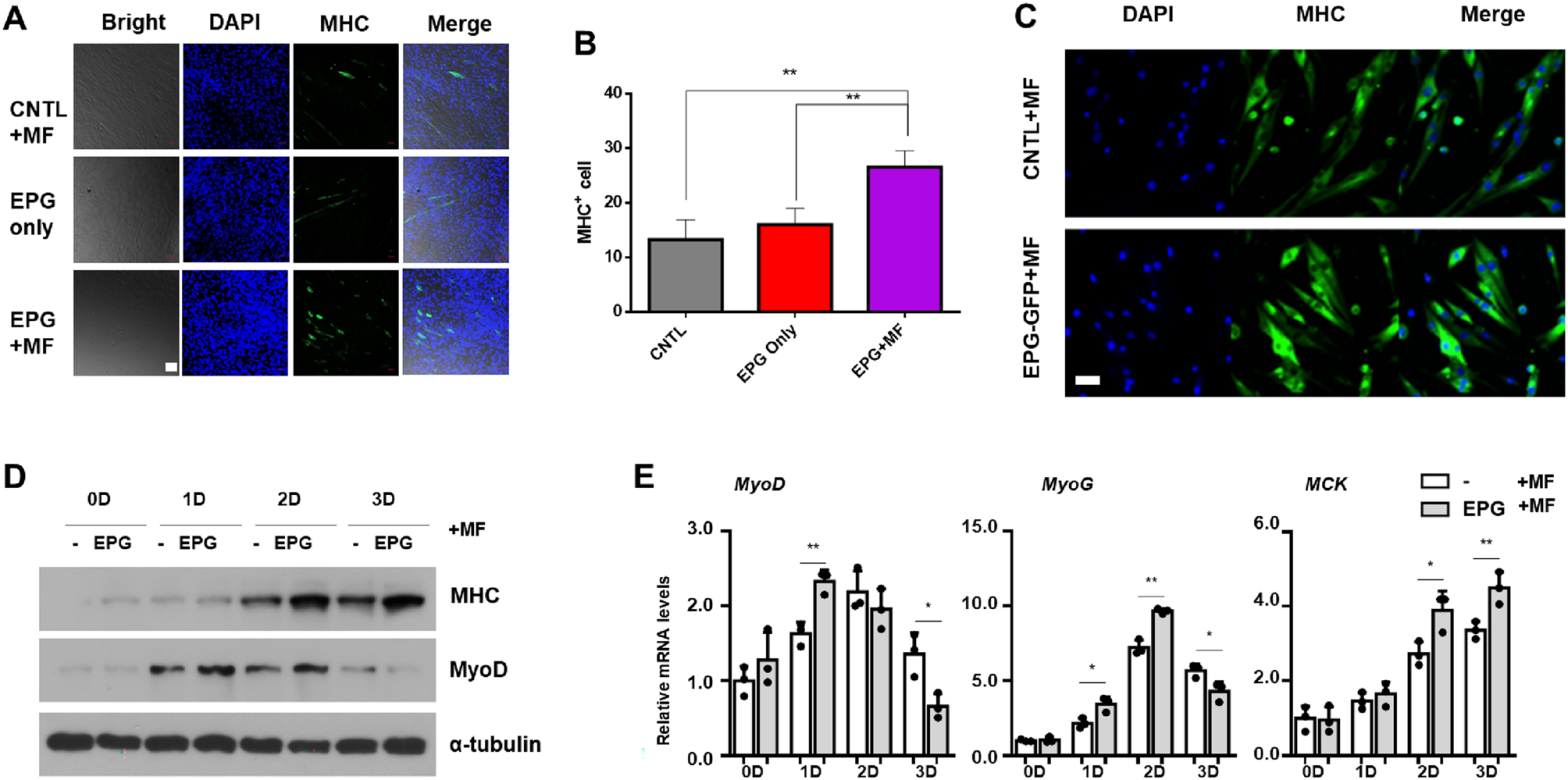
Myogenic differentiation of EPG transfected muscle primary cells. (**A**) Immunostaining of MHC positive C2C12 cells (MHC^+^, green; scale bar = 20 µm; MF = ~ 196 mT for 24 h). (**B**) Analyzed graph from A. (**C**) Immunostaining of MHC^+^ satellite cells (MF = ~ 196 mT for 24 h, scale bar = 20 µm, DAPI, FITC: 488/520). (**D**) Western blot of MHC, MyoD, and reference gene in EPG-satellite cells. (**E**) Relative mRNA expression of EPG-satellite cells (MF = ~ 196 mT for 24 h; cultured 0–3 d).

Myoblast differentiation is a multistep process that depends on [Ca^2+^]i and allows for the activation of transcription factors and Ca^2+^-dependent kinases or phosphatases^28, 29^. Antigny et al.^29^ suggested that higher levels of Ca^2+^ induced and promoted myocyte differentiation more. C2C12 cells were transfected with EPG and treated with MF at day 2 for 24 h and then differentiated with 2% horse serum for another 7 days. After immunostaining for myotube (MHC), the EPG + MF group showed enhanced in the MHC^+^ cells (28 ± 3 cells per field), whereas the EPG only and control groups displayed levels of 14 ± 4 and 18 ± 3 cells per field (*P* < 0.001), respectively (Fig. 4A,B).

Skeletal muscle satellite cells are quiescent mononucleated myogenic cell. These cells are normally quiescent in adult muscle, but increase their population and proliferation in response to injury^30^. Therefore, we also evaluated of satellite cells when EPG was expressed. We isolated satellite cells from mouse tibialis anterior and gastrocnemius muscles and transfected with EPG-RFP (Supporting Fig. 3B). Same as C2C12 cells, satellite cells were transfected with EPG and treated with MF at day 2 for 24 h and then differentiated with 2% horse serum for another 5 days. EPG + MF group, MHC^+^ cells showed increase of 30% fluorescence intensity against control group (Fig. 4C). It also proved in immunoblotting assay that MHC and MyoD were more expressed with EPG + MF in Fig. 4D. Similar to C2C12 results, mRNA expression was elevated 1.7-fold (MyoD) at day 1, 1.3-fold (MyoG) at day 2, and 1.5-fold increase (MCK) at day3 with EPG + MF treatment respectively (Fig. 4E), which play major roles in regulating muscle differentiation. We confirmed that EPG + MF had a greater influence on myogenesis, which may compose the body muscle.

### EPG in mice

**Figure 5:**
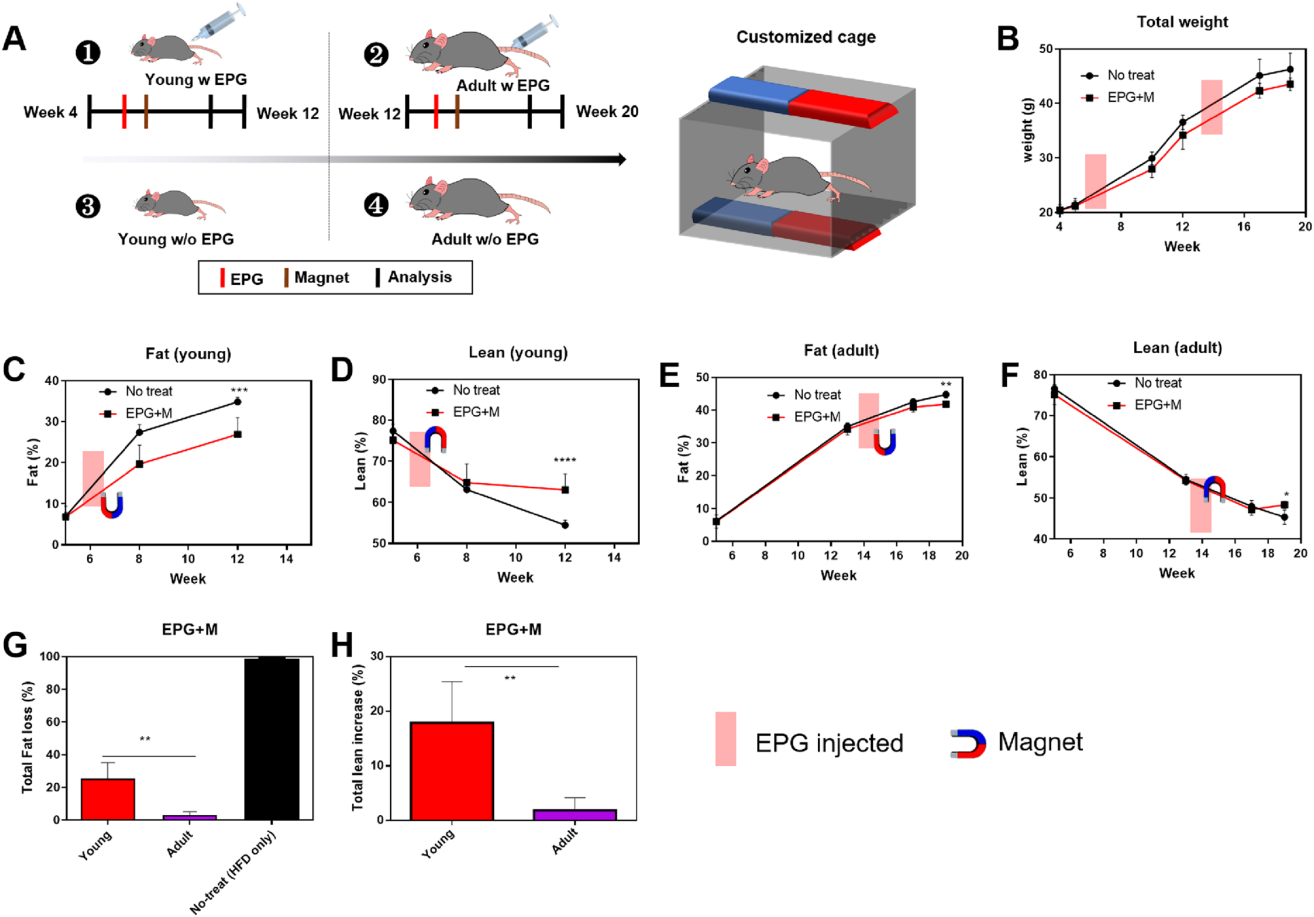
Weight, fat, lean mass analysis of EPG-injected mice. (**A**) Illustration of the experimental setup (left), Customized cage set up (right, 6 × 10 × 6 cm, Neodymium magnet were placed both sides- 50 × 20 × 5 mm, ~ 196 mT, 30 min). (**B**) Total body weight (AAV6-EPG was injected two times). (**C**) Total fat analysis of young mice (AAV6-EPG was injected at week 6). (**D**) Total lean analysis of young mice (AAV6-EPG was injected at week 6). (**E**) Total fat analysis of adult mice (AAV6-EPG was injected at week 14). (**F**) Total lean analysis of adult mice (AAV6-EPG was injected at week 14). (**G**) Fat accumulation of young and adult mice (HDF = no-treated group). (**H**) Lean development of young and adult mice (AAV6-EPG = 3 × 10^10^ Pa; Magnetic field was applied for 5 d; n = 5).

Regulation of transcription factors (Ca^2+^) and certain mRNA by EPG with a MF results in the differentiation of more myoblasts and formation of fewer fat droplets in adipocytes. Fat formation or acculumation could be different on age. We tested these hypothesis in an in vivo animal models. Thirty mice were fed a 60% HFD for 20 weeks including control group (Fig. 5A), following which they were divided into four groups: young no-treat group (4 week-old, fed until week 13), young EPG group (4 week-old, fed until week 13) and adult no-treat group (12 week-old, fed until week 20), and adult EPG-group (12 week-old, fed until week 20, n = 6). Body fat and lean weight, blood components, total weight, and histological changes were observed in each group. To verify EPG expression in the body, AAV6-EPG-RFP was injected into the mouse thigh at week 5, and in vivo imaging was performed using IVIS^®^ for 5 d to observe a weak RFP signal (Supporting Fig. 6A).

In the experimental groups, AAV-6 EPG-RFP (3 × 10^10^ Pa, 50 μL) was injected into young and adult mice biceps femoris at weeks 6 and 14. Mice were placed in a customized cage (6 × 10 × 6 cm) and MF (~ 196 mT) was applied to them for 30 min on 5 consecutive days.

We found that there was a continuous increase in total body weight during the experiment due to the administration of HFD to the mice; however, the EPG with a MF-treated group showed 10% less total weight after 2 weeks of EPG injection (Fig. 5B, Table 2).

**Table 2:** Individual total weight, fat, lean mass change.

Week	No treat					EPG treat				
	Number	Total weight	Fat	Fluid	Lean	Number	Total weight	Fat	Fluid	Lean
Week 1	N1	27.9	4.1	1.03	20.72	EPG1	26.6	4.06	1.12	20.83
	N2	27.3	4.06	1.05	20.4	EPG2	27.9	4.12	1.12	20.83
	N3	27.1	3.92	1.08	20.3	EPG3	26	3.72	10.48	19.45
	N4	26.9	3.93	1.04	20.25	EPG4	25.2	3.18	1.018	18.99
	N5	DEAD				EPG5	26.2	3.55	1.08	16.2
EPG injected										
Week 2	N1	34.5	10.64	1.43	21.48	EPG1	30.72	7.822	1.19	19.612
	N2	35.66	10.64	1.4	21.5	EPG2	28.9	5	1.28	20.75
	N3	33.54	9.177	1.17	21.3	EPG3	30.78	8.46	1.26	18.97
	N4	34	5.7	1.25	20.44	EPG4	28.53	6.57	1.23	18.87
	N5					EPG5	29.45	5.99	1.31	19.92
Week 4	N1	39.57	16.3	1.2	20.7	EPG1	38	13.94	1.29	20.39
	N2	41.47	16.96	1.377	21.73	EPG2	34.78	9.28	1.2	22.02
	N3	38.47	13.77	1.25	21.53	EPG3	36.95	13.27	1.32	19.45
	N4	41.88	16.78	1.419	21.38	EPG4	32.8	10.1	1.19	19.22
	N5	DEAD				EPG5	34.8	10.299	1.306	20.89
	N1	42.15	18.1	1.22	19.8	DEAD				
	N2	46.4	20.32	15.32	21.16	EPG2	41.95	14.77	1.46	22.38
Week 6	N3	42.8	17.74	1.377	20.44	EPG3	44.27	17.92	1.55	20.73
	N4	46	19.74	1.56	20.99	EPG4	38.4	14.1	1.47	19.47
	N5	DEAD				EPG5	42.25	16.02	1.579	20.916
	N1	44.1	19.2	1.47	20.76	DEAD				
	N2	48.63	21.848	1.74	22.05	EPG2	45.15	18.047	1.74	22.7
Week 8	N3	44.81	18.99	1.65	21.52	EPG3	47.97	20.67	1.728	22.625
	N4	47.5	20.439	1.997	22.06	EPG4	42.37	17.4	1.759	20.467
	N5	DEAD				EPG5	44.88	18.8	1.87	21.39
	N1	47.31	21.05	1.3	22.58	DEAD				
	N2	46.3	22.02	1.418	21.133	EPG2	50.4	22.668	1.685	23.86
Week 13	N3	50.79	22.419	1.716	24.1	EPG3	49.13	20.905	1.846	24.234
	N4	49.93	21.81	1.87	23.96	EPG4	49.27	22.379	1.841	22.837
	N5	DEAD				EPG5	46.2	21.13	1.746	23.139

There were no significant changes in alanine transaminase and aspartate aminotransferase levels between the young and adult groups. However, the adult group showed higher enzyme levels owing to HFD (Supporting Fig. 6B). On the other hand, both the EPG + MF-treated young and adult groups showed lower total cholesterol levels (20 ± 2% and 15 ± 1%, respectively, *P* < 0.05), while there were no changes in the TG levels. The glucose levels increased in young mice (25 ± 1%) treated with EPG + MF. Upon treatment with EPG with a MF, HDL levels decreased in adult mice (30 ± 3%), whereas LDL levels decreased (70 ± 5%) in young mice only (Supporting Fig. 6C). After two weeks of EPG + MF treatment, fat loss and muscle gain were observed in the young mice group. Total fat decreased by 25 ± 8% (Fig. 5C), while total lean increased by 18 ± 7% at the 12th week, as compared to that in the untreated group (HFD only group) (Fig. 5D). The EPG + MF-treated adult group showed a similar pattern of less fat (− 6 ± 2%) (Fig. 5E) and more lean (6 ± 3%) in the 19th week (Fig. 5F). When the feeding time, MF treatment, and amount of EPG-expressing virus transfected were equal, more myogenesis and less adipogenesis were observed in the young mouse group than that in the adult group (*P* < 0.001 and *P* < 0.05, respectively) (Fig. 5G,H).

### Observation of EPG-treated mice

**Figure 6:**
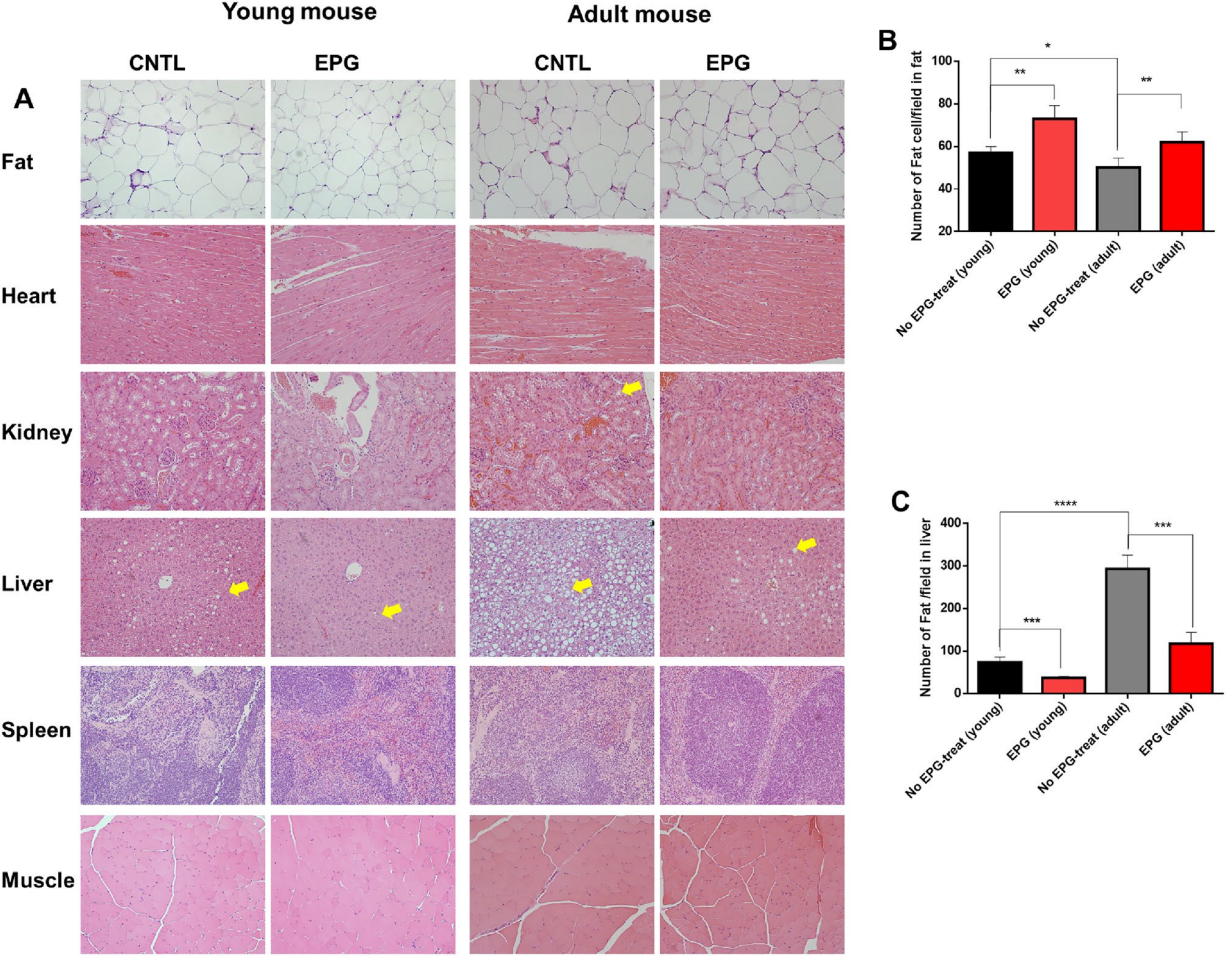
Hematoxylin and Eosin (H&E) staining of EPG-injected mice. (**A**) H&E staining of six organs (Fat, liver, Heart, kidney, Spleen, and Muscle). (**B**) Fat cell count per field in abdominal fat samples. (**C**) Lipid count per field in liver samples (mice were scarified in 7 weeks after EPG injection, AAV6-EPG = 3 × 10^10^ Pa; Magnetic field (customized cage) was applied for 30 min–5 days; n = 3; yellow arrows indicate fat).

WE performed qPCR after EPG treatment in mice. GAPDH, MHC1 (*P* < 0.0001), MyoD, and MyoM (*P* < 0.05) were increased while MyoG showed no change in Supporting Fig. 7. Next, six organs (heart, liver, spleen, muscle, fat, and kidney) were harvested at the 13th and 20th weeks, following which the morphological changes in them were observed using hematoxylin & eosin staining (Fig. 6A). Owing to intake of HFD, the adult mouse group displayed an increase in the fat volume against no EPG-treat group (Fig. 6A, top); interestingly, the EPG + MF-treated group displayed a smaller increase in fat volume in comparison. In both cases, the EPG + MF-treated group showed higher fat density (*P* < 0.001) (Fig. 6B) and lower fat droplet accumulation in liver (*P* < 0.0001) (Fig. 6C). There were no morphological differences in the hearts, spleens, or muscles of the two groups. Thus, decreased adipogenesis occurred in both mice under the regulation of [Ca^2+^]i by EPG + MF.

Although both the young and adult mouse groups showed different levels of anti-adipogenetic and myogenetic effects, there were clear protein (mRNA) activity restraining and arresting effects that were mediated via [Ca^2+^]i upon EPG with a MF, thereby indicating a regulatory role of Ca^2+^ in adipogenesis and myogenesis.

In summary, EPG with magnetic field prevented fat accumulation and enhanced muscle development; this effect was more significant in the younger mice. Therefore, EPG could be used as a molecular and therapeutic agent against childhood obesity, instead of traditional methods such as a combination of diet, exercise, and/or behavior modifications.

## Discussion

Ca^2+^ plays an important role in cell homeostasis, function, duplication, and differentiation^31^. In this study, we focused on the role of [Ca^2+^]i and their ability to differentiate primary cells into adipocytes and myocytes by EPG with a MF. Although, mechanism of EPG in regulation of Calcium influx is unclear, however different level of cellular calcium level serves as transcriptional regulators in many cells.

In this study, Differentiation-related genes, such as PPARγ, GLU4, MyoD, and MyoG, were evaluated to determine the role of the sudden change in [Ca^2+^]i upon EPG with a MF. The present study provide evidence indicating the regulatory role of Ca^2+^ in adipogenesis and myogenesis.

First, there were drastic morphological changes, including fewer lipid droplets in adipose cells and greater number of MHC^+^ cells in myocytes that received EPG with a MF. Second, the decreased GAPDH activity in the group that received the EPG only and EPG with a MF treatments also served as evidence for the inhibitory effect of [Ca^2+^]i. Third, gene expression levels of CasR, PPARγ, and GLU4 reduced, while those of MyoG and MyoD increased, due to EPG with a MF-mediated regulation of [Ca^2+^]i. It has been shown that GLU4 induces adipogenesis by stimulating the entry of glucose into cells, under the influence of insulin or PPARγ2^32^, and MyoG and MyoD, which bind to the regulatory regions of a battery of skeletal muscle genes and activate their transcription during muscle differentiation^33^. Finally, upon EPG with a MF treatment, anti-adipogenetic and myogenetic effects were observed in the HFD-fed animals with regulation of total body and lean fat.

HFD has influenced both EPG-treated and non-EPG-treated group. However, the EPG + MF-treated group showed 10% less total weight after 2 weeks. We also discovered that total fat accumulation and lean increase were different between the young and adult groups. The young mice group showed higher total fat decreased by 25 ± 8% comparing to adult group (− 6 ± 2%), (Fig. 5C,E) that EPG may have more potential effect against obesity in developing ages. On the other hand, the young group gained 18 ± 7% total lean, while adult group gained a similar, but less, amount of lean. (6 ± 3%). Thus, EPG + MF is more effective in the young group than the adult group.

We tested long term investigation EPG on obesity model in second in vivo experiment (32 week). Total fat decreased 25 ± 10% in the EPG group after 2 weeks of EPG injection, which was similar to the young EPG-treated group. However, total fat was regained as much as control group (HDF only) by week 10 (Supplementary Fig. 8) then, sustained for another 20 weeks. It indicates that EPG is a temporary and repeatable treatment against obesity. In addition, further investigation is necessary on the toxic issue, duration, and EPG in both the chow diet model or and the diabetic model.

## Material and methods

### Reagents

Lipofectamine™ 2000, Lipofectamine™ 3000, Fura-4AM, Hank’s balanced salt solution (HBSS), PureLink™ RNA Mini Kit, tris(2-carboxyethyl), and GAPDH assay kit were purchased from Thermo Fisher (Seoul, Korea). The cDNA and qPCR master mixes were purchased from Cellsafe (Yong-in, Korea). All DNA oligonucleotides were purchased from Bioneer (Daejeon, Korea) and subjected to high-performance liquid chromatography purification. Magnetic beads (2–2.9 μM) were purchased from Spherotech (Illinois, USA). All other chemicals and reagents were purchased from Sigma-Aldrich (Seoul, Korea), except if specifically mentioned.

### Cell maintenance

HEK-293T, C2C12 cells, and satellite cells were a kind gift from Prof. Hong’s laboratory (Department of Life Sciences, School of Life Sciences and Biotechnology, Korea University). Human adipose-derived stem cells (hADSCs) were purchased from Cefobio (Kwang-myeong, Korea). All cells were used between passages 3 and 10 and maintained in Dulbecco’s modified Eagle medium (DMEM) or Ham’s/F-10 with additional supplements, if required. All media were supplemented with 1% antibiotics (penicillin 100 U/mL and streptomycin 100 µg/mL) and heat-inactivated 10% fetal bovine serum in a 5% CO_2_-containing atmosphere at 37 °C.

### Apply magnetic field on cells

To activate EPG, either neodymium magnet were placed under the 96 well (50 × 20 × 5 mm, ~ 196 mT) for 12 h to 48 h according to purpose or 10 µL of magnetic beads (~ 50 mT) was added directly into culture dish. All control groups were treated MF same as sample groups unless otherwise stated. For in vivo animal study, mice were placed in a customized cage (6 × 10 × 6 cm) and MF (~ 196 mT) was applied to them for 30 min on 5 consecutive days.

### Generation of EPG-expressing plasmids

pcDNA3.1-EPG- and pcDNA3.1(+)-c-Fos-(GGS) × 6-HaloTag C58 were purchased from Addgene (Watertown, MA, USA). pcDNA3.1-EPG-GFP and -RFP were cloned and constructed at Bionics (Seoul, Korea).

### Adeno-associated virus (AAV) production

The AAV particle for EPG-RFP expression was generated using the AAVpro^®^ Helper-Free System (Takara Bio). The pAAV6-EPG-RFP vector was constructed by cloning the EPG-RFP-coding region of pcDNA3.1-EPG-RFP into the multiple cloning site of pAAV6-CMV, according to the manufacturer’s protocol. AAVpro^®^ 293T cells (Seoul, Takara Korea) were seeded on a 150-mm dish and transfected with AAV plasmid constructs using the TransIT-VirusGEN^®^ Transfection Reagent (MIR6700, Wisconsin, Mirus Bio). AAV particles were isolated from the cell pellets using the AAV Extraction Solution (Takara Bio), and the AAV viral titer was determined using the AAVpro^®^ Titration Kit (Takara Korea).

### qRT-PCR analysis for target sequences

Cells were seeded on a 6-well plate at a density of 2 × 10^4^ cells/mL and cultured to 80% confluence. After transfection with EPG-RFP, the cells were treated with a magnet for 24 h, following which the medium was replaced with differentiation medium and the cells were cultured in it for 7–10 days. The cells were then harvested for total RNA extraction using the PureLink™ RNA Mini Kit (Thermo Fisher Scientific). RNA (500 ng) was reverse-transcribed using the cDNA Master Mix (Cellsafe). Real-time qPCR was performed on cDNA using the qPCR master mix (Cellsafe). Primers were purchased from Bioneer (Supporting Table 1). The mRNA expression was normalized to ATP5PB (β-actin) or GAPDH expression using the 2^−∆∆Ct^ method. Each experiment was performed twice, in duplicate.

### Cytotoxicity test

C2C12 cells and hADSCs were cultured in a 96-well plate and then transfected with EPG when they reached a confluency of 80%. After 24 h of transfection, a MF was applied to the cells for 10 h and 24 h respectively. Next, 100 μL of a 1:20 diluted CCK-8 solution (CCK-8 diluted in DMEM, v/v) was added to each well, and the cells were incubated with it for 4 h. The percentages of live and dead cells were spectrophotometrically analyzed at the wavelength of 450 nm.

### EPG transfection

HEK-293T cells, C2C12 cells, and hADSCs plated in a 24-well dish were transfected with pcDNA3.1-EPG-GFP, pcDNA3.1-EPG-RFP, only RFP, or only GFP, using Lipofectamine™ 2000 or 3000, according to standard protocols. The transfected cells were incubated for 48–60 h at 37 °C, following which the GFP- and RFP-expressing cells were confirmed using a fluorescence microscopy.

### Co-transfection of EPG and C-fos-RFP

HEK-293T cells plated in a 24-well dish were co-transfected with pcDNA3.1-EPG-GFP and C-fos-RFP or RFP alone using Lipofectamine™ 2000, according to standard protocols. The transfected cells were incubated for 48 h at 37 °C, and RFP-expressing cells were imaged before and after the addition of magnetic beads, using a EVOS M5000 system (Thermo Fisher).

### Differentiation of cells

C2C12 cells were cultured in 10% fetal bovine serum-containing DMEM and then differentiated in 2% horse serum-containing DMEM, for 7 days. hADSCs were cultured in ADSC growth medium (Cefobio) and differentiated in differentiation medium (Cefo), for 10 days.

### Satellite cell preparation and culture

The tibialis anterior and gastrocnemius muscles of eight-week old C57BL/6 mice were injected with 1 or 4 μg of cardiotoxin (CTX), respectively. After 3 days, damaged muscles were isolated from the mice and incubated with 250 CDU/mL of collagenase from Clostridium histolyticum (Sigma, C6885) for 30 min at 37 °C. Single muscle fibers were collected by triturating the muscle tissue using a Pasteur pipette in Ham’s/F-10 nutrient mixture (Welgene, LM 009-01) containing 20% horse serum, 100 units/mL penicillin, and 100 μg/mL streptomycin. The harvested muscle fibers were transferred to a new conical tube, centrifuged at 300 g for 1 min at room temperature, and the supernatant was discarded. The muscle fiber pellets were re suspended in growth medium [Ham’s/F-10 nutrient mixture containing 20% horse serum, 5 ng/mL recombinant human FGF-basic (Peprotech, 100-18B), 100 units/mL penicillin, and 100 μg/mL streptomycin] and plated in Matrigel matrix (Corning, 354234)-coated dishes. Next day, the suspended muscle fibers were removed and cells attached to the Matrigel matrix-coated dishes were passaged using PBS to obtain satellite cells. Later, Satellite cells plated in 6-well dishes were transfected with pcDNA3.1-EPG-RFP using Lipofectamine™ 2000 for further experiments.

### Satellite cell differentiation

To differentiate satellite cells into myotubes, satellite cells were cultured in DMEM containing 2% horse serum for 5 days.

### C2C12 differentiation staining

After 7 d of differentiation, the cells were fixed and permeabilized with 4% paraformaldehyde and 1% Triton™ X-100. Next, the cells were blocked with 2% bovine serum albumin and treated with 1:500 diluted MF20 (DSHB). After washing 2 times, the cells were treated with 1:2000 diluted secondary antibody (FITC-conjugated anti-mouse antibody, ABclonal), for 16 h.

### Intracellular calcium mobilization

Calcium imaging was performed on HEK-293T, C2C12 cells and hADSCs transfected with pcDNA3.1-EPG-GFP or -EPG-RFP using Lipofectamine™ 2000 or 3000. The cells were imaged at 48 h post-transfection, following which they were washed thrice with HBSS. Next, the cells were loaded with 1 µM Fura-4AM in HBSS for 30 min at 37 °C, following which they were washed twice with HBSS. The Fura-4AM in the cells was then de-esterified for 30 min at 37 °C. The level of cytoplasmic Ca^2+^ was estimated in terms of the signal intensity at the excitation/emission wavelengths of 490/520, using a EVOS M5000 system. The changes in signal intensity before and after magnetic field from beads were analyzed using ImageJ software^34^.

### Lipid staining

Oil-red O staining of lipid droplets (red) was followed by hematoxylin staining (purple, for the nucleus). Briefly, hADSCs were transfected and stimulated with a magnet for 24 h and then cultured for 10 d. Next, the cells were washed and fixed with 4% formalin, incubated in 60% isopropanol (for 5 min), and immersed in Oil Red O solution (0.3% Oil Red O dissolved in 0.18% isopropanol). To counterstain the cells, a drop of hematoxylin was used for 1 min. Finally, the stained cells were washed several times for clarity and examined under an inverted light microscope (CX30, Olympus, Japan).

### Immunoblot analysis

After transection of EPG in satellite cells, cell lysates were prepared using TNE lysis buffer (20 mM Tris–HCl, 150 mM NaCl, 2 mM EDTA, 1% NP-40, 50 mM NaF, 1 mM Na-orthovanadate, and protease inhibitors). Denaturated cell lysates were separated on an SDS–polyacrylamide gel and transferred to PVDF membranes. The membranes were incubated at 4 °C overnight with antibodies (primary 1:100, secondary 1:2000) targeting various proteins (MYH1E Antibody (MF 20)—DSHB, MyoD (NovusBio, NB100-56511), and α-tubulin (Santa Cruz Biotechnology,sc-5286) (Supporting Fig. 9).

### GAPDH activity assay

The GAPDH assay was performed on the cell lysates. ADSCs were transfected and stimulated with a magnet for 24 h, following which the cells were harvested, by means of centrifugation at 14,000 × *g*. The assay was performed by addition of 100 μL ice-cold GAPDH assay buffer to the cell pellet, followed by the steps outlined in the manufacturer’s protocol (Abcam, Cambridge, UK).

### TG content assay

After differentiation by means of EPG and MF, the TG content was quantified using a TG POCT kit (Barozen, Korea). Briefly, ADSCs were plated in a 6-well plate, at a cell density of 5 × 10^5^ cells/mL and cultured, followed by transfection, magnetic field, and differentiation. Post this, the cells and supernatants were harvested. The cell lysates were collected by means of centrifugation (14,000 × *g* at 4 °C, for 10 min) and then heated at 80 °C, for 5 min, following which the TG levels in 50 μL of lysate and supernatant were measured as per the manufacturer’s instructions.

### Generation of the obesity mouse model

All animal experiments were approved by the Institutional Animal Care and Use Committee of Korea University, Korea (KOREA-2021-0204). Animal handling and experiments were performed in strict accordance with the guidelines and recommendations of the Institutional Animal Care and Use Committee of Korea University. Additionally, the present study was carried out in compliance with the ARRIVE guidelines.

Four-week-old female C57/BL6J mice were obtained from OrientBio (Seong-Nam, Korea). The mice were housed in an animal care facility, under ambient conditions of temperature and relative humidity, for a week. The six groups of mice were fed a high-fat diet (HFD; 60% fat per kcal). The mice were maintained on this diet for 19 weeks, following which their body weight, total fat, and total lean weight were measured before and after MF treatment.

### Fat and lean analysis

Briefly, C57BL/6J mice were anaesthetized using alfaxalone (30–60 mg/kg). Alfaxalone was diluted at a ratio of 1:10 with phosphate-buffered saline and 0.1 mL of it was injected intramuscularly into the mice. Measurements were performed according to the provided protocol. Total fat and lean fat contents were measured by Lean / Fat Analyzer (Minispec LF90II) in the 5th, 8th, 12th, 17th, and 20th weeks.

### EPG treatment and organ harvest

The mice were divided into young and adult groups. The mice in the young group were injected with AAV6-EPG (50 μL, 3 × 10^10^ Pa) at the back of their thighs in the 6th week. After 4 d of resting, the mice were treated with a MF (~ 196 mT) in a customized cage for 5 d. The mice in the adult group were injected with AAV6-EPG (50 μL, 3 × 10^10^ Pa) at the back of their thighs in the 14th week, following which a MF was applied. The mice were sacrificed 7 weeks after EPG injection, following which six of their organs (liver, spleen, kidney, heart, abdominal fat muscle, and blood) were harvested for further experiments.

### Gene analysis after EPG treatment in mice and long term investigation

Second set of mouse experiment was performed on 4 week-old mice for 32 weeks (n = 5). All set-up was same as first experiment. Mice were sacrificed after EPG + MF treatment for 5 day then, biceps femoris was collected homogenized and isolated mRNA for RT-PCR (MyoG, MyoD, Myomaker, MHC1, and GAPDH).

### Statistical analysis

Prism software (GraphPad) was used for statistical analysis and graphical representation of the data. Student’s *t*-tests were performed to evaluate statistical significance. Unless otherwise stated, data are presented as mean ± standard deviation or percentage of mean. To assess statistical significance, analysis of variance with post-hoc analysis was performed using a web-based statistics calculator (http://astatsa.com/OneWay_Anova_with_TukeyHSD/).

### Supplementary Information

Supplementary Information 1.

Supplementary Information 2.

## Data Availability

The authors declare that all data needed to support the findings of this study are presented in this article or in the Supporting Information.
