# Author Correction: Regulation of myogenesis and adipogenesis by the electromagnetic perceptive gene

**DOI:** 10.1038/s41598-024-57169-w

**Published:** 2024-03-19

**Authors:** Jangsun Hwang, Hae Woon Jung, Kyung Min Kim, Daun Jeong, Jin Hyuck Lee, Jeong-Ho Hong, Woo Young Jang

**Affiliations:** 1 Department of Orthopedic Surgery, College of Medicine, Korea University, 73 Korea-ro, Seongbuk-gu, Seoul, 02841 Republic of Korea; 2 Department of Pediatrics, Kyung Hee University Medical Center, Seoul, Republic of Korea; 3 Department of Life Sciences, School of Life Sciences and Biotechnology, Korea University, Seoul, 02841 Republic of Korea; 4 Institute of Nano, Regeneration, and Reconstruction, College of Medicine, Korea University, 73 Korea-ro, Seongbuk-gu, Seoul, 02841 Republic of Korea

Correction to: *Scientific Reports* 10.1038/s41598-023-48360-6, published online 01 December 2023

The original version of this Article contained an error in the spelling of the authors Hae Woon Jung and Jin Hyuck Lee which was incorrectly given as HaeWoon Jung and JinHyuck Lee.

The original Article has been corrected.

