# Supplementary material for: Regulation of myogenesis and adipogenesis by the electromagnetic perceptive gene
Source: Sci Rep. 2023 Dec 1;13:21167. doi: 10.1038/s41598-023-48360-6 (PMC10689489; doi:10.1038/s41598-023-48360-6)
Supplement: Supplementary file 1 — Supplementary Information 1. [file 41598_2023_48360_MOESM1_ESM.docx]

**Supplementary information**

Title: Regulation of myogenesis and adipogenesis by the electromagnetic perceptive gene

Jangsun Hwang^ad†^, Hae Woon Jung^b†^, Kyung Min Kim^c^, Daun Jeong^ad^, Jin Hyuck Lee^ad^, Jeong-Ho Hong^c^, and Woo Young Jang^ad*^

^a^Department of Orthopedic Surgery, College of Medicine, Korea University, 73 Korea-ro, Seongbuk-gu, Seoul 02841, Republic of Korea

^b^Department of Pediatrics, Kyung Hee University Medical Center, Seoul, Korea

^c^Department of Life Sciences, School of Life Sciences and Biotechnology, Korea University, Seoul 02841, Korea

^d^Institute of Nano, Regeneration, Reconstruction, College of Medicine, Korea University, 73 Korea-ro, Seongbuk-gu, Seoul 02841, Republic of Korea

†These authors contributed equally.

^*^Correspondence to opmanse@korea.ac.kr


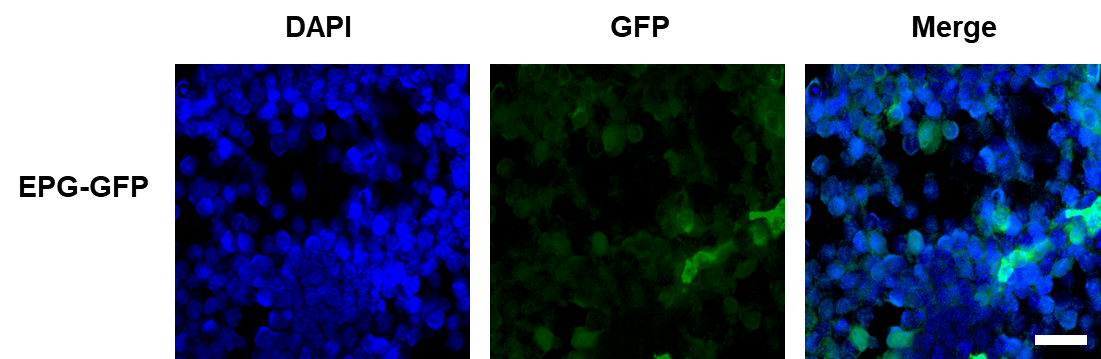


**Supporting Figure 1**. **Fluorescent images of EPG-GFP transfected HEK 293T cells** (scale bar =50 µm, DAPI: 358⁄461 nm, GFP: 475/510 nm).


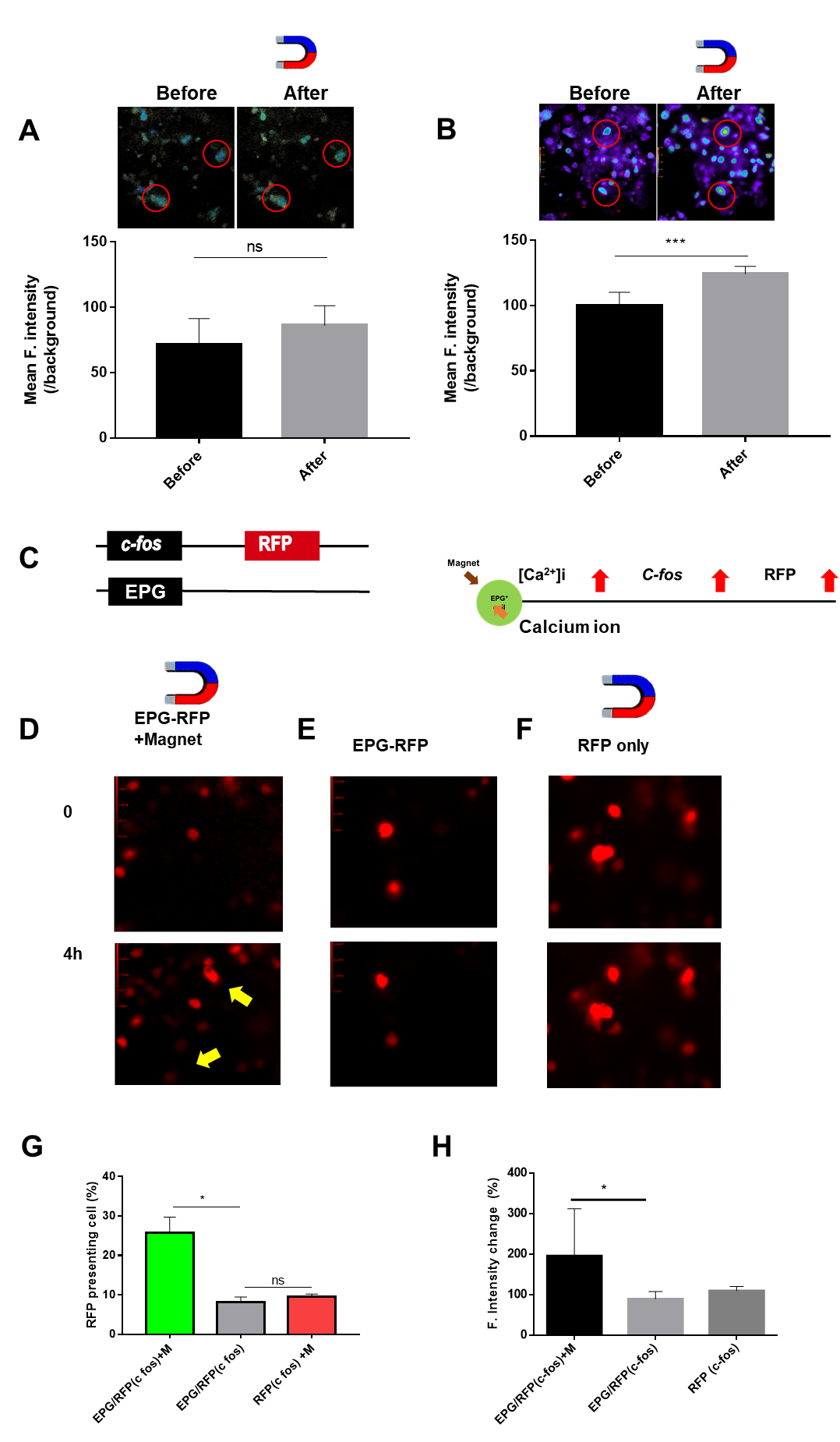


**Supporting Figure 2**. **Calcium ion mapping and** **regulation of RFP expression by EPG with calcium ion sensitive promotor on HEK 293T cells. (A)** Calcium ion mapping on HEK 293T cells without magnet. **(B)** EPG-GFP transfected HEK 293T cells with magnet (HEK 293T cells were transfected with EPG and stained with Fura-2AM, florescence intensity was measured individual cell before and after magnetic bead application, Magnetic field=50mT). **(C)** Illustration of target gene regulation on EPG-GFP and c-fos-RFP co transfected HEK 293T cell. **(D)** Fluoresce images of EPG, c-fos-RFP co transfected HEK 293T cell without magnet. **(E)** Fluoresce images of c-fos-RFP transfected HEK 293T cell with magnet. **(F)** RFP transfected HEK 293T cell with magnet. **(D)** Quantification of RFP expression from D-F **(H)** Analyze the intensity of RFP expression from D-F (HEK 293T cells were observed and countered after 4h in the presence of magnetic beads, 50 mT).


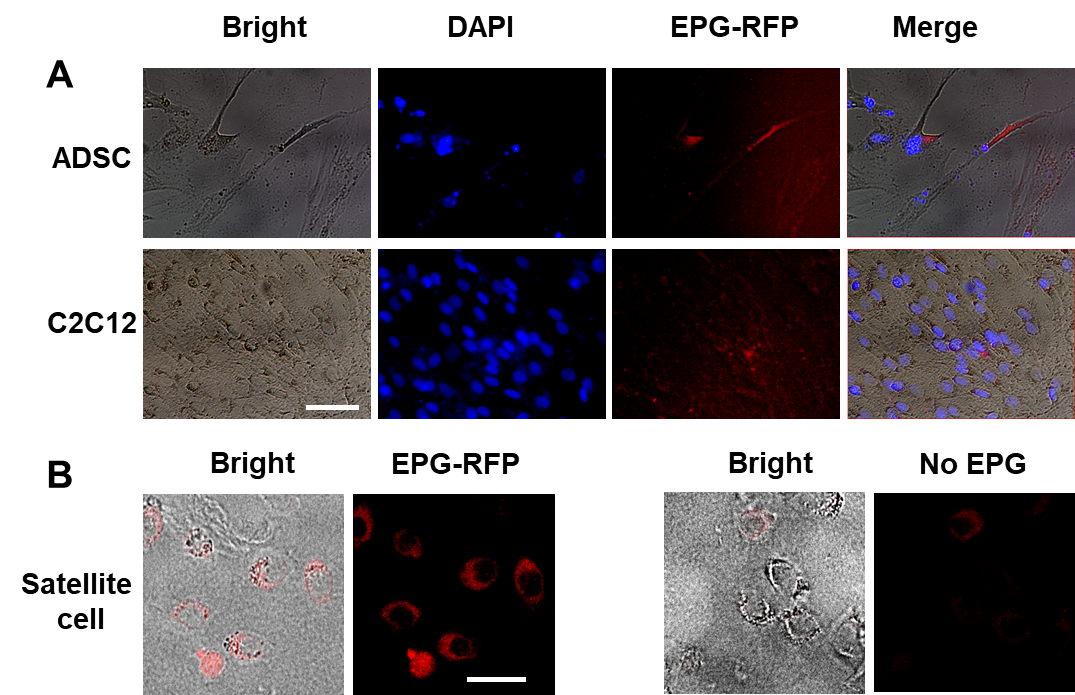


**Supporting Figure 3**. **Fluorescent images of EPG transfected (A) ADSC, C2C12 cells, and (B) satellite cells** (scale bar =20 or 50 µm, DAPI: 358⁄461 nm, EPG-RFP: 540/585 nm).


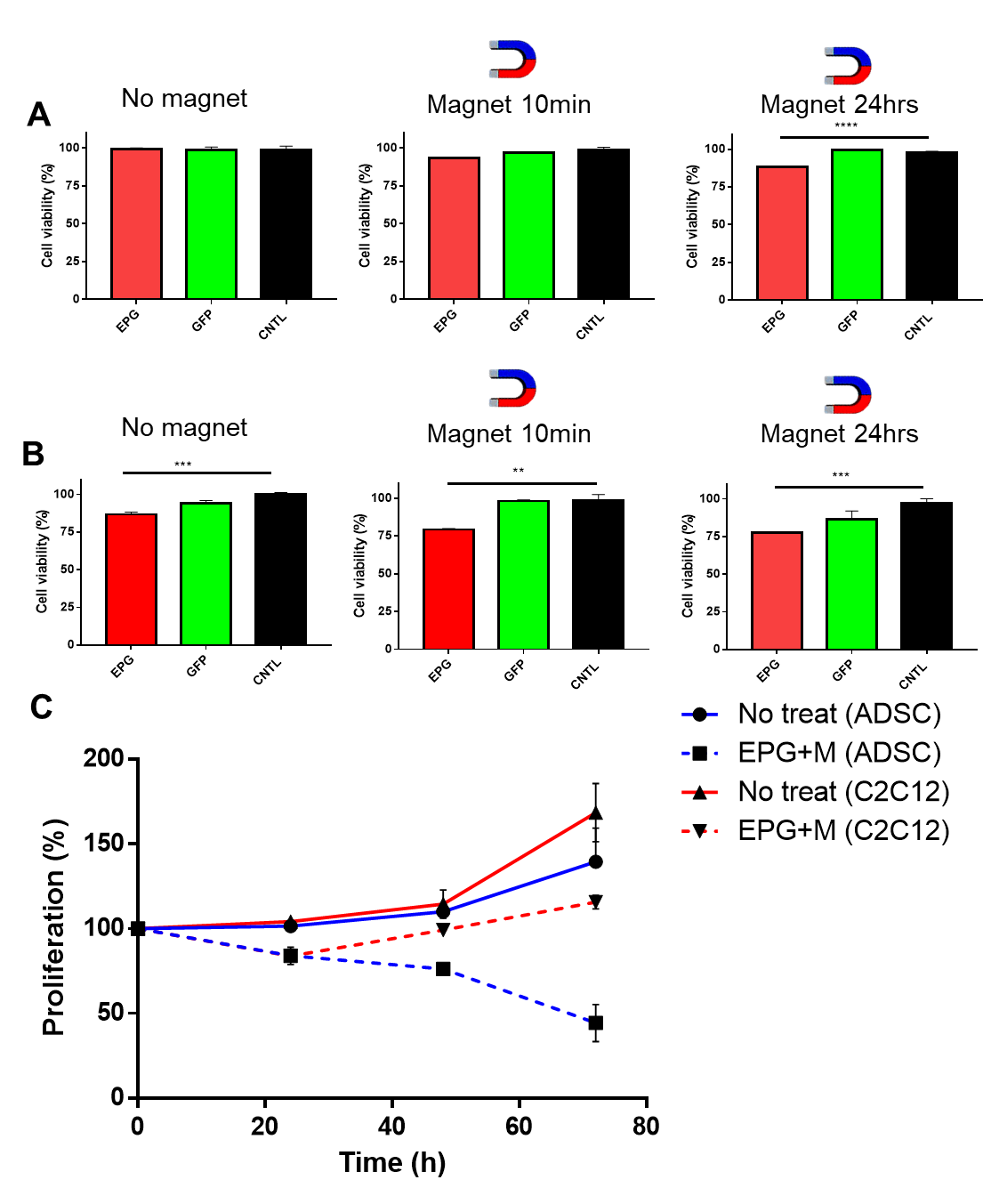


**Supporting Figure 4. Proliferation test of EPG on ADSCs and C2C12**. **(A)** EPG transfected ADSCs without magnet stimulation, magnet for 10 min, and 24h (left to right). **(B)** EPG transfected C2C12 cells without magnet stimulation, magnet for 10 min, and 24h (left to right). **(C)** Cell viability (magnet= ~196mT, CNTL=no treat, n=3, 5x 10^4^/mL cells were used and cultured for 24-72 h, CCK-8 assay).


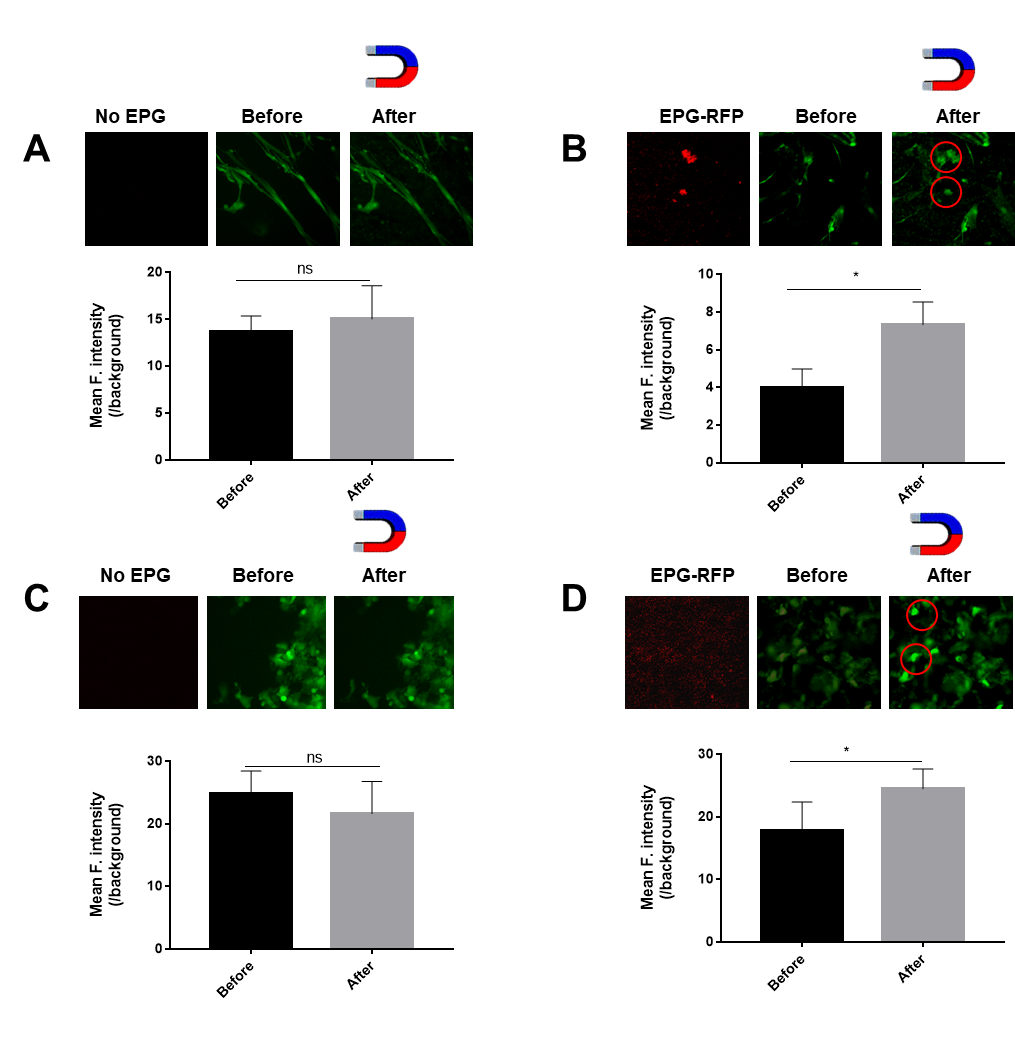


**Supporting Figure 5. Calcium ion mapping. (A)** [Ca2+]i images on ADSC without EPG activation. **(B)** [Ca2+]i images on hADSC with EPG with magnetic activation. **(C)** [Ca2+]i images on C2C12 without EPG activation**. (D)** [Ca2+]i images on C2C12 with EPG with magnetic activation (EPG-RFP=red, magnetic bead = ~196mT, Green=Fura -4AM, red circle= Ca^2+^ increased cell, n=3).


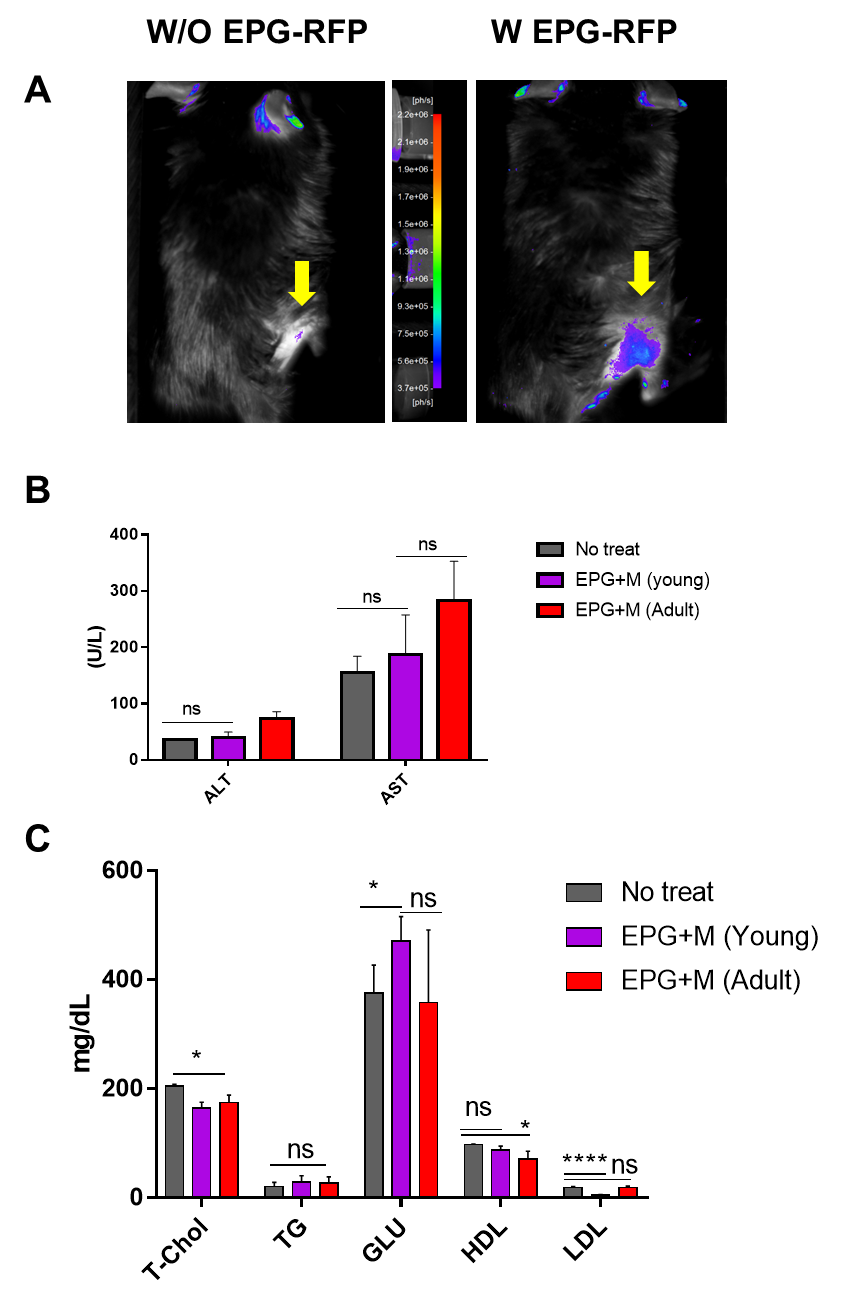


**Supplementary Figure 6**. **Evaluation of EPG in mouse. (A)** IVIS image of EPG-RFP expression on a mouse. **(B)** ALT and AST. **(C)** Lipid profile test (all mice were treated MF with customized cage, AAV6-EPG-RFP= 3x10^10^ Pa, magnetic field ~196mT, 30 min for 5 days, n=3).


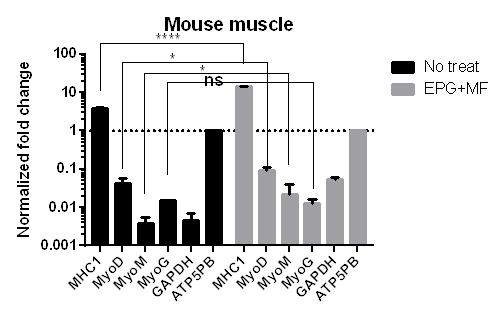


**Supplementary Figure 7**. **Gene evaluation after** **EPG treat.** (EPG injected mice were treated with MF in customized cage for 5 day and sacrificed , AAV6-EPG-RFP= 3x10^10^ Pa, magnetic field ~19 6mT, 30 min for 5 days, n=3).

**Supplementary Figure 8**. **Long term investigation EPG on obesity model.** (4 weeks-old mouse, EPG injected at week 2, HFD fed until 32 week, AAV6-EPG-RFP= 3x10^10^ Pa, magnetic field ~19 6mT, 30 min for 5 days, n=5).


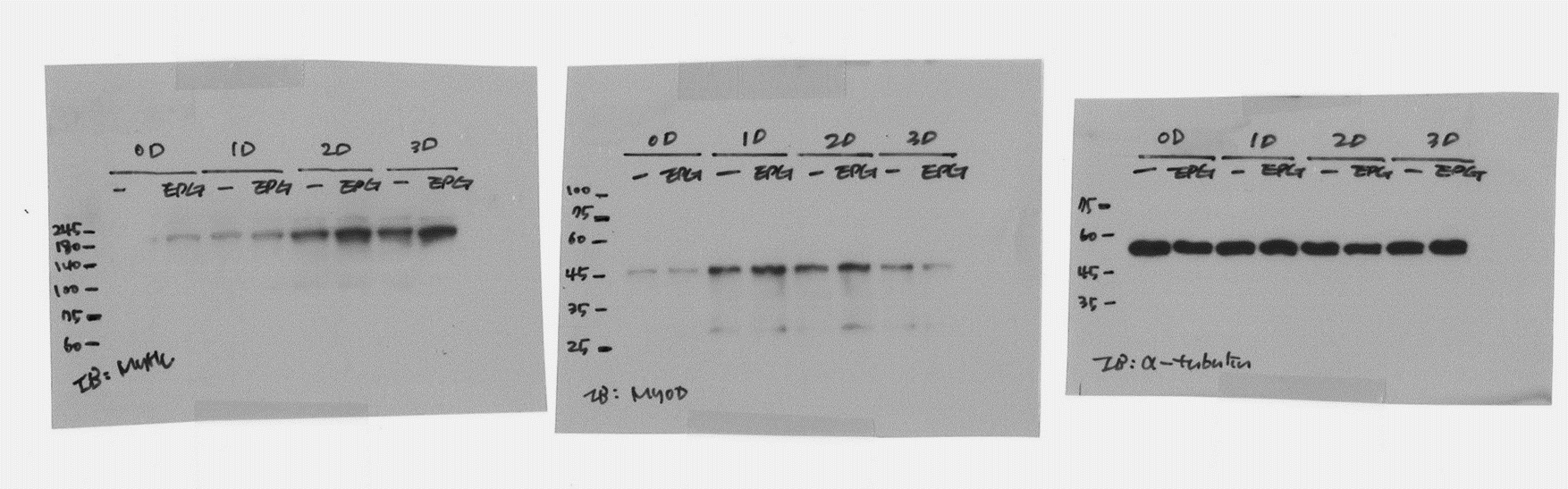


**Supplementary Figure 9**. **Uncropped full-length pictures of western blotting membranes.** Full-length blot image for figure 4D (MHC, MyoD, and α-tubulin, primary antibody= 1:100, secondary antibody= 1:2000) targeting various proteins (MYH1E Antibody (MF 20) – DSHB, MyoD (NovusBio, NB100-56511), and α-tubulin (Santa Cruz Biotechnology,sc-5286, HRP-conjugated anti-mouse antibody was used, Membranes were often cut to enable blotting for multiple antibodies).

**Table 1**. **Primer pairs**

| **Protein** | **Gene** | **Forward** | **Reverse** |
| --- | --- | --- | --- |
| Murine MyoD | MyoD | 5’ GCCGGTGTGCATTCCAA 3’ | 5’ CACTCCGGAACCCCAACAG 3’ |
| Murine MyoG | MyoG | GACCTGATGGAGCTGTATGAG | CTGAAGGTGGACAGGAAGG |
| Murine Myomaker | MYMK | CTGAGCTCCCAAGACATGAG | CCAATCTCTCCTTCCTCTGG |
| Murine MCK | MCK | CACCTCCACAGCACAGACAG | ACCTTGGCCATGTGATTGTT |
| Murine MHC | MHC1 | ACAAGCTGCGGGTGAAGAG | CAGGACAGTGACAAAGAACG |
| Murine GAPDH | GAPDH | GTTGTCTCCTGCGACTTCA | GGTGGTCCAGGGTTTCTTA |
| Human PPARγ2 | PPARγ2 | CTATTGACCCAGAAAGCGAT | CGTAATGTGGAGTAGAAATGC |
| Human GLUT4 | GLUT4 | AGGATCGGTTCTTTCATCTTCGC | GTTCCCCATCTTCGGAGCCTA |
| Human CasR | CasR | TCTCAAATCAAGGCCGGAGT | GCTGTTTATCTCCTCTATGGCAA |
| Human Beta actin | ATP5PB | TCGAGTCGCGTCCACC | GGGAGCATCGTCGCCC |

**Table 2. Long term investigation of EPG and Individual total weight, fat, lean mass change**

|  |  |  | No treat | |  |  |  |  | EPG treat | |  |
| --- | --- | --- | --- | --- | --- | --- | --- | --- | --- | --- | --- |
| week | Number | Total weight | Fat | Fluid | Lean |  | Number | Total weight | Fat | Fluid | Lean |
| week 1 | N1 | 27.9 | 4.1 | 1.03 | 20.72 |  | EPG1 | 26.6 | 4.06 | 1.12 | 20.83 |
|  | N2 | 27.3 | 4.06 | 1.05 | 20.4 |  | EPG2 | 27.9 | 4.12 | 1.12 | 20.83 |
|  | N3 | 27.1 | 3.92 | 1.08 | 20.3 |  | EPG3 | 26 | 3.72 | 10.48 | 19.45 |
|  | N4 | 26.9 | 3.93 | 1.04 | 20.25 |  | EPG4 | 25.2 | 3.18 | 1.018 | 18.99 |
|  | N5 | DEAD | | | |  | EPG5 | 26.2 | 3.55 | 1.08 | 16.2 |
| **EPG injected** | | | | | | | | | | | |
| week 2 | N1 | 34.5 | 10.64 | 1.43 | 21.48 |  | EPG1 | 30.72 | 7.822 | 1.19 | 19.612 |
|  | N2 | 35.66 | 10.64 | 1.4 | 21.5 |  | EPG2 | 28.9 | 5 | 1.28 | 20.75 |
|  | N3 | 33.54 | 9.177 | 1.17 | 21.3 |  | EPG3 | 30.78 | 8.46 | 1.26 | 18.97 |
|  | N4 | 34 | 5.7 | 1.25 | 20.44 |  | EPG4 | 28.53 | 6.57 | 1.23 | 18.87 |
|  | N5 |  |  |  |  |  | EPG5 | 29.45 | 5.99 | 1.31 | 19.92 |
|  |  |  |  |  |  |  |  |  |  |  |  |
| week 4 | N1 | 39.57 | 16.3 | 1.2 | 20.7 |  | EPG1 | 38 | 13.94 | 1.29 | 20.39 |
|  | N2 | 41.47 | 16.96 | 1.377 | 21.73 |  | EPG2 | 34.78 | 9.28 | 1.2 | 22.02 |
|  | N3 | 38.47 | 13.77 | 1.25 | 21.53 |  | EPG3 | 36.95 | 13.27 | 1.32 | 19.45 |
|  | N4 | 41.88 | 16.78 | 1.419 | 21.38 |  | EPG4 | 32.8 | 10.1 | 1.19 | 19.22 |
|  | N5 | DEAD | | | |  | EPG5 | 34.8 | 10.299 | 1.306 | 20.89 |
|  |  |  |  |  |  |  |  |  |  |  |  |
|  | N1 | 42.15 | 18.1 | 1.22 | 19.8 |  | DEAD | | | | |
|  | N2 | 46.4 | 20.32 | 15.32 | 21.16 |  | EPG2 | 41.95 | 14.77 | 1.46 | 22.38 |
| week 6 | N3 | 42.8 | 17.74 | 1.377 | 20.44 |  | EPG3 | 44.27 | 17.92 | 1.55 | 20.73 |
|  | N4 | 46 | 19.74 | 1.56 | 20.99 |  | EPG4 | 38.4 | 14.1 | 1.47 | 19.47 |
|  | N5 | DEAD | | | |  | EPG5 | 42.25 | 16.02 | 1.579 | 20.916 |
|  |  |  |  |  |  |  |  |  |  |  |  |
|  | N1 | 44.1 | 19.2 | 1.47 | 20.76 |  | DEAD | | | | |
|  | N2 | 48.63 | 21.848 | 1.74 | 22.05 |  | EPG2 | 45.15 | 18.047 | 1.74 | 22.7 |
| week 8 | N3 | 44.81 | 18.99 | 1.65 | 21.52 |  | EPG3 | 47.97 | 20.67 | 1.728 | 22.625 |
|  | N4 | 47.5 | 20.439 | 1.997 | 22.06 |  | EPG4 | 42.37 | 17.4 | 1.759 | 20.467 |
|  | N5 | DEAD | | | |  | EPG5 | 44.88 | 18.8 | 1.87 | 21.39 |
|  |  |  |  |  |  |  |  |  |  |  |  |
|  | N1 | 47.31 | 21.05 | 1.3 | 22.58 |  | DEAD | | | | |
|  | N2 | 46.3 | 22.02 | 1.418 | 21.133 |  | EPG2 | 50.4 | 22.668 | 1.685 | 23.86 |
| week 13 | N3 | 50.79 | 22.419 | 1.716 | 24.1 |  | EPG3 | 49.13 | 20.905 | 1.846 | 24.234 |
|  | N4 | 49.93 | 21.81 | 1.87 | 23.96 |  | EPG4 | 49.27 | 22.379 | 1.841 | 22.837 |
|  | N5 | DEAD | | | |  | EPG5 | 46.2 | 21.13 | 1.746 | 23.139 |
