# Supplementary figures and images for: Regulation of myogenesis and adipogenesis by the electromagnetic perceptive gene
Source: Sci Rep. 2023 Dec 1;13:21167. doi: 10.1038/s41598-023-48360-6 (PMC10689489; doi:10.1038/s41598-023-48360-6)

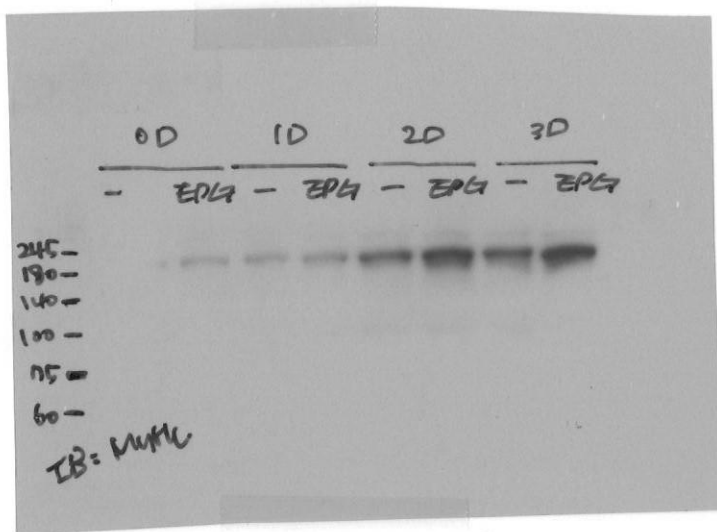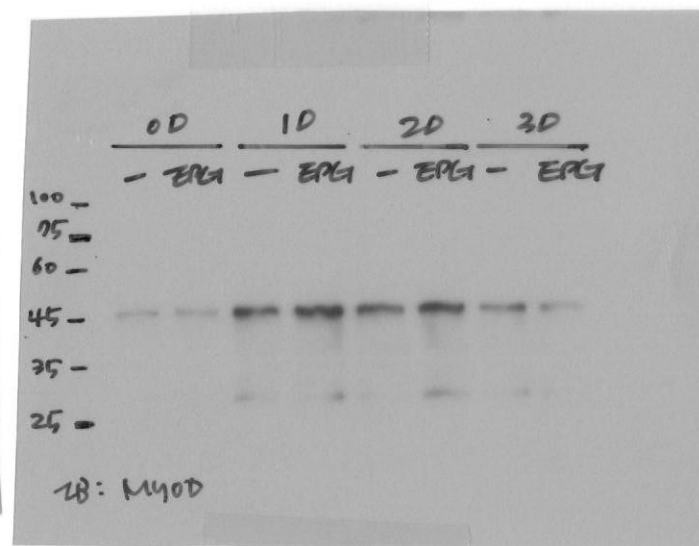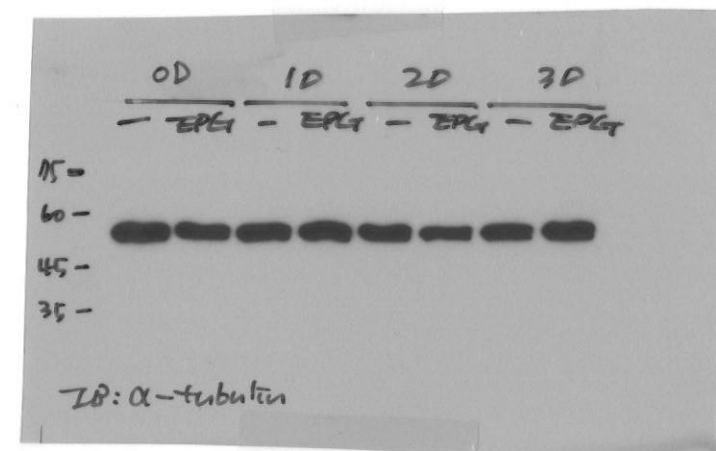

Supplement: Supplementary file 2 — Supplementary Information 2. [file 41598_2023_48360_MOESM2_ESM.pdf]
